# Measuring self-reported ability to perform activities of daily living: a Rasch analysis

**DOI:** 10.1186/s12955-021-01880-z

**Published:** 2021-10-18

**Authors:** Eva Ejlersen Wæhrens, Anders Kottorp, Kristina Tomra Nielsen

**Affiliations:** 1 The ADL Unit, The Parker Institute, Copenhagen University Hospital Bispebjerg-Frederiksberg, Nordre Fasanvej 57, 2000 Frederiksberg, Denmark; 2 Occupational Science and Occupational Therapy, User Perspectives and Community-Based Research, Department of Public Health, University of Southern Denmark, J. B. Winsløwsvej 9a, 5000 Odense, Denmark; 3 Faculty of Health and Society, Malmö University, 20506 Malmö, Sweden; 4 Department of Occupational Therapy, University College of Northern Denmark, Selma Lagerløfsvej 2, 9220 Aalborg, Denmark

## Abstract

**Background:**

Since the number of persons diagnosed with multi-morbidity is increasing, there is a need for generic instruments to be able to assess, measure and compare ADL ability across diagnoses. Accordingly, the ADL-Interview (ADL-I) was developed to be used in rehabilitation research and clinical practice. The aim of this study was to investigate if the ADL-I can be used to provide valid and reliable ADL ability measures across gender and diagnostic groups.

**Methods:**

ADL-I data were extracted from an existing research database on persons with chronic conditions including medical, rheumatological, oncological, neurological, geriatric and psychiatric diagnoses. Data were analysed based on Rasch Measurement methods to examine: the psychometric properties of the rating scale; ADL item and person fit to the Rasch model; if the difficulty of the ADL tasks differs across gender and diagnostic groups, and if the ADL-I provides precise and reliable measures of ADL ability.

**Results:**

Data on n = 2098 persons were included in the final analysis. Initial evaluation of the 0–3 rating scale revealed threshold disordering between categories 1 and 2. After removal of 16 underfitting items, the variance explained by the Rasch dimension increased from 54.3 to 58.0%, thresholds were ordered, but the proportion of persons with misfitting ADL-I measures increased slightly from 8.7 to 9.1%. The person separation index improved slightly from 2.75 to 2.99 (reliability = 0.90). Differential test function analysis, however, supported that the 16 underfitting items did not represent a threat to the measurement system. Similarly, ADL items displaying differential item functioning across gender and diagnoses did not represent a threat to the measurement system. The ADL items and participants were well distributed along the scale, with item and person measures well targeted to each other, indicating a small ceiling effect and no floor effect.

**Conclusions:**

The study results overall suggest that the ADL-I is producing valid and reliable measures across gender and diagnostic groups among persons within a broad range of ADL ability, providing evidence to support generic use of the ADL-I.

**Trial registration:**

N/A.

## Background

All human beings have a need to perform activities of daily living (ADL). A need perceived by the person and/or by society. ADL includes both Personal ADL (PADL) and Instrumental ADL (IADL). PADL tasks are typically performed on a daily basis, regardless of gender, housing conditions, culture and interests. PADL cover tasks such as toileting, bathing, dressing, and eating. IADL involves tasks that are necessary to live an independent life and cover more complex tasks including cleaning, shopping, and cooking [1]. The ability to perform ADL tasks are often affected by acute, long-term or chronic conditions. Thus, problems related to ADL are typically targeted in the rehabilitation process [2]. To plan and implement rehabilitation interventions it is therefore necessary to evaluate the clients’ initial level of ADL ability.

Within the field of rehabilitation, several instruments used to evaluate ADL ability have been developed. Some are diagnosis–specific, others are generic i.e., for use across gender and diagnostic groups. The Barthel Index [3] and the Functional Independence Measure (FIM™) [4] are examples of generic instruments that are well-known and commonly used within rehabilitation. In both instruments the ability to perform PADL tasks independently is evaluated based on an ordinal rating scale and the interpretation is based on summed scores. ADL ability, however, also involves performance of the more complex IADL tasks and need of help is only one of several aspects related to performance. Hence, decreased quality of ADL task performance may also be indicated by increased effort, inefficient use of time and safety risk.

The Assessment of Motor and Process Skills (AMPS) [5, 6] is an example of a generic observation-based instrument covering both PADL and IADL. Further, the instrument is developed to measure the quality of ADL task performance based on several aspects including physical effort, efficiency, safety and independence. The AMPS is developed based on Rasch measurement methods and when using the instrument, linear measures of both ADL motor and ADL process ability are generated based on an interval scale. The psychometric properties of the observation-based AMPS ADL ability measures have been established across gender and diagnostic groups [7]. Still, observation-based measures represent the outsider’s perspective which has limited relationship to the insider’s perspective i.e., the person’s perceived ability [8–10]. Until recently, an ADL instrument, based on self-report, focused on the quality of performance and providing linear measures, was not available. Consequently, the ADL-Interview (ADL-I) was developed [8, 11].

The ADL-I is an instrument developed to describe and measure the quality of ADL task performance based on self-report. When administering the ADL-I, persons are asked to evaluate the quality of their performance in 47 specific ADL tasks; 31 tasks related to PADL and 16 tasks related to IADL. The aspects of quality of performance evaluated in the ADL-I are similar to the aspects evaluated in the AMPS: physical effort, efficiency, safety and independence. ADL-I data can be used to generate an overall linear measure of self-reported quality of ADL task performance. Similar to the AMPS, Rasch measurement methods have been employed to develop the ADL-I [11]. So far, the ADL-I has been applied in research studies among various diagnostic groups e.g. rheumatic diseases [8], depression [9], chronic obstructive pulmonary disease (COPD) [12], advanced cancer [10, 13], a mixed sample of chronic conditions [14] and in a geriatric population [15]. For each of these research studies, study-specific ADL-I measures have been generated based on Rasch measurement methods, verifying validity and reliability of the measures. Since the ADL-I was developed as a generic instrument to also be used in clinical rehabilitation settings, representing a need to measure ADL ability across a diverse group of patients including patients with multimorbidity, the next step is to investigate if the ADL-I can be used to provide valid and reliable ADL ability measures across gender and diagnostic groups. Based on previous research [16] we did expect to find some variation in difficulty of ADL tasks across gender and diagnostic groups, but not to the point that it would affect the measurement system.

## Methods

### Aim

The overall aim was to examine the psychometric properties of the ADL-I applied among males and females living with various chronic conditions. More specifically, the following research questions were addressed.

- Does a four-category rating scale used with the ADL tasks (items) of the ADL-I demonstrate sound psychometric properties?
- Do the ADL tasks (items) define a single unidimensional construct?
- Do the person responses demonstrate expected and valid response-pattern?
- Does the difficulty of the ADL tasks (items) differ across gender and diagnostic groups?
- Does the ADL-I provide precise and reliable measures of ADL ability?

### Design and setting

The study was a descriptive register-based study. All data was obtained from an existing research database at the ADL unit of the Parker Institute, Copenhagen University Hospital, Bispebjerg og Frederiksberg. The database contains data on self-reported ADL task performance using the ADL-I collected in a range of research studies since 2007 by occupational therapists, trained in administering the ADL-I. The database contains no client identifiers besides diagnosis, gender and age. Hence, anonymized data from various client groups is included in the database, e.g., persons with rheumatologic diseases, cancer, COPD, schizophrenia, depression, mild stroke and persons with geriatric or orthopaedic problems.

### Participants/materials

Self-reported ADL ability reflects the person’s perceived ability and is based on experiences of performance. Persons with decreased ADL ability due to acute illness will have very limited experience with their present ability to perform ADL task. Accordingly, only ADL-I data on diagnostic groups considered to be living with chronic conditions were included. Chronic conditions were defined by Goodman et al. as “*conditions that last a year or more and require ongoing medical attention and/or limit activities of daily living*” [17]. Hence, data on persons with medical, rheumatological, oncological, neurological, geriatric and psychiatric chronic conditions were extracted from the abovementioned database. Subsequently, persons with maximum scores were excluded. In order to characterize the study sample, demographic data (gender and age) was also extracted from the database.

### Instrumentation

The ADL-Interview (ADL–I) [18] is an occupational therapy evaluation tool developed to describe and measure the quality of ADL task performance based on self-report (i.e. ADL ability). When administering the ADL-I, persons are asked to evaluate the quality of their performance in 47 ADL tasks; 31 tasks related to PADL and 16 tasks related to IADL. The ADL tasks are defined are organised into 12 ADL domains; *Eating and drinking, Mobility, Going to the toilet, Dressing, Personal Hygiene, Grooming, Communication, Transportation, Cooking, Shopping, Cleaning* and *Washing*, based on the ADL Taxonomy [19, 20].

When reporting the quality of ADL task performance, the person uses seven response categories: (a) I perform the task independently without the use of extra time or effort and without risk; (b) I perform the task independently without the use of extra time or effort and without risk, but I use helping aids; (c) I perform the task independently, but it takes me extra time; (d) I perform the task independently, but I use extra effort/get tired faster; (e) I perform the task independently, but there is a risk that I might hurt myself; (f) I need assistance from someone, but I do participate; and (g) The task is performed by others for me—I cannot participate actively. For clinical purposes, the persons can use more than one response category if several apply to their performance of the specific ADL task. If a person finds a task irrelevant to his or her daily life, the response category “Not relevant” is used.

Evaluation of the quality of PADL task performance is based on the past 24 h, whereas quality of IADL task performance is based on the past week. ADL-I data can be used to describe task-specific self-reported quality of ADL task performance in a single person or a group of persons as well as to generate an overall measure of self-reported quality of ADL task performance. To generate overall linear measures, the mark given in the lowest response category on each task for each person is rated using a four-point ordinal rating scale: *Competent* (score = 3) covering response categories (a) and (b), *Using extra time/effort* (score = 2) covering response categories (c) and (d), *Need for help/safety* (score = 1) covering response categories (e) and (f), and *Unable* (score = 0) covering response category (g). The ordinal scores are then, based on the Rasch rating scale model, transformed into an overall linear (interval scale) measure of self-reported quality of ADL task performance, adjusted for the difficulty of the ADL tasks.

The ADL-I was initially developed and validated in persons with various types of rheumatic diseases [8]. Later studies have supported the validity of the ADL-I to describe and measure self-reported quality of ADL task performance in persons with other chronic conditions e.g. depression [9], COPD [12] and incurable cancer [10, 13]. Further, a study suggested that the ADL-I is sensitive to change in ADL ability among older persons participating in a reablement program [15].

### Data analyses

The Rasch computer program, WINSTEPS version 4.7.0 [21] was used to convert ordinal scores into equal interval units or measures of the person’s self-reported overall quality of ADL task performance. The conversions are based on log-odds probabilities; thus, the item difficulty and the quality of ADL task performance measures are expressed in logits (log-odds probability units) [22]. The measures of item difficulty, and quality of ADL task performance, respectively, represent item and person location along the linear scale. WINSTEPS was also used to generate several statistics to evaluate aspects of validity and reliability, including fit of the data to the Rasch model assertions [23, 24]. An overview of the analysis is provided in Table 1. Details related to Rasch analysis procedures have been described elsewhere [23–25].

**Table 1:** Overview of the Rasch analysis

Steps in the analysis	Procedures	Indicators/criteria
1. Selecting a Rasch Measurement model	Evaluation of the log likelihood ratio	A non-significant (p > 0.05) log likelihood ratio indicates that data fits an interval scale model i.e. the Rasch Rating Scale Model
2. The psychometric properties of the ADL-I rating scale	Following Linacre’s guidelines [28–30]	Frequency distribution across response categories should be either uniform or peak in central or extreme categories to illustrate optimal use of the categories
		Average category measures should advance monotonically up the rating scale, indicating that persons, who experience higher quality of performance, have higher item ratings
		Scale category outfit mean square (*MnSq*) values should be ≤ 2.0
		Threshold calibrations should advance monotonically, with no threshold disordering
		Thresholds should increase by at least 1.4 logits to show distinction between categories, but by no more than 5 logits to avoid large gaps in the variable [29, 30]
3. Principal Component Analysis (PCA)	Identification of possible secondary dimensions within the data	The proportion of variance explained by the measure must be > 50% The largest secondary dimension should have an eigenvalue < 2.0 (i.e. less than two items) to support unidimensionality [33]
	Examination of potential secondary dimensions: division of ADL-I items into three clusters based on item loadings, estimation of a measure for each person on each cluster and performance of Pearson correlations between measures	A disattenuated correlation (correlation based on measures adjusted for their standard error) > 0.7 between clusters would support unidimensionality [33]
4. Item goodness-of-fit	Examining infit and outfit statistics. Items displaying underfit misfit were removed one at the time, in the order of highest *MnSq* values, considering high infit *MnSq* values first	*MnSq* values between 0.7 and 1.3 logits, combined with *z* values ≥ 2.0, indicated item fit [34]
	Removal of underfitting items was planned to stop when all items met the criteria for acceptable goodness-of-fit	Assuming the PCA does not support the presence of a secondary dimension in the data, an instrument is generally considered to be unidimensional, when no more than 5% of the items fail to fit the Rasch model (p < 0.05) [32]
5. Person goodness-of-fit	Evidence of person-response validity was evaluated by examining the person goodness-of-fit statistics	The criterion for acceptable person goodness-of-fit was infit *MnSq* values < 1.3 logits associated with a z value of < 2.0 [35, 36] It was accepted that, by chance, up to 5% of the sample would fail to demonstrate acceptable goodness-of-fit without a serious threat to validity [36, 37]
6. After removal of misfitting items	Persons with maximum scores on this shorter version were removed, and analyses of rating scale properties, PCA and person goodness-of-fit repeated	Determine if scale properties and unidimensionality had improved
7. Differential Item Functioning (DIF)	Determine if item difficulty estimates vary across gender and diagnostic groups	An item was considered to display DIF, when the difference in item difficulty estimates between groups was > 0.50 logits [38] and statistically significant (p < 0.01) [33, 39, 40]
8. Differential Test Functioning (DTF)	Scatterplots of the variance of person ability measures across versions were produced	A criterion was set that no more than 5% of the participants should differ significantly (z-values exceeding ± 1.96) between the two measures [41]
9. Reliability and precision	Determine if the mean item difficulty measure was appropriately targeted to the mean person ability measure	The mean person ability measure would be close to zero for a well-targeted instrument [23]
	Examining the item-person map	Dispersion of item difficulty and person ability measures were evaluated for a reasonable match
	Precision was evaluated by overall separation and reliability indices	Separation indices should be at least 2.0 to obtain a desired reliability coefficient of 0.80 for replicability of person ability and item difficulty ordering [42] The closer the reliability index was to 1.0 (range 0.0 to 1.0) the better [43]

Within Rasch measurement methods, the Partial Credit Model (PCM) [26] and the Rasch Rating Scale Model (RSM) [27] are applied with data derived from response scales with more than two categories. The only difference between the two models is related to their assumptions about distance between the response categories. The PCM assumes that the distance between the response categories is not the same, whereas the RSM assumes equal distances between categories. Evaluation of the log likelihood ratio indicated fit to an interval model (*p* = 0.3548), thus, the RSM was applied. The RSM for ADL-I includes two facets (items and persons) and is based on two assertions: (a) a person experiencing more quality of ADL task performance is more likely to receive higher ratings on harder ADL-I items than a person experiencing lower quality of ADL task performance; and (b) any person is more likely to receive higher ratings on easier ADL-I items than on harder ADL-I items [23]. When data meet these expectations, the items and the persons fit the measurement model, supporting internal scale and person response validity of the ADL-I, respectively.

Prior to the RSM analysis, persons with maximum scores on ADL-I were removed, since they mathematically correspond to infinite or indefinite measures on the latent variable and so are not directly estimable [25]. To address the first research question, Linacre’s guidelines [28–30] for evaluation of the psychometric properties of a rating scale was applied. The second research question, related to determine if items in the ADL-I represent a single unidimensional construct, was addressed in several analyses including a Principal Component Analysis (PCA) of the standardized residuals (i.e. the difference between what the Rasch model predicts and what was observed), item and person goodness-of-fit and Differential Item Functioning (DIF) for gender and diagnostic groups [31, 32]. Moreover, since the clinical relevance of the tasks (items) included in the ADL-I has already been verified in previous studies [19, 20], it was investigated whether the measurement system would be disrupted if clinically relevant, but misfitting items and/or items displaying DIF were retained. This was done by evaluating for Differential Test Functioning (DTF) [31].

The PCA of the standardized residuals was performed to identify possible secondary dimensions within the data. The analyses of goodness-of-fit to the Rasch model included both infit and outfit statistics. While the infit statistics are more sensitive to unexpected patterns of observations on items that are roughly targeted to the people, outfit statistics are more sensitive to unexpected observations on items that are very easy or very hard [33]. Also, both underfit and overfit to the model was identified [23]. While underfit degrades the quality of measures, overfit in general has no practical implications, but might be an indication of lack of local independence (i.e. significant correlations among the items after the contribution of the underlying construct is removed). Therefore, items displaying overfit misfit were not considered a threat to the measurement system and retained in the instrument. Subsequently, the investigation of unidimensionality was continued by evaluation of person-response validity and analyses of DIF. DIF occurs when item difficulty estimates vary between groups, thus representing a risk to the unidimensionality requirement. To determine if the ADL-I can be used as a generic tool to measure self-reported quality of ADL task performance, DIF was evaluated based on gender and diagnosis.

When items displaying misfit had been identified and removed, persons with maximum scores on this shorter version of ADL-I were removed, and analyses of the psychometric properties of the rating scale, PCA, person goodness-of-fit and DIF were repeated, to determine if scale properties and unidimensionality had improved.

Afterwards, analyses of DTF were performed to determine if relevant, but removed, items would disrupt the measurement system if kept in the instrument. DTF occurs when person ability measures vary between two versions of a test. The evaluation of DTF related to inclusion or omission of misfitting ADL items was performed by comparing (a) measures of quality of ADL task performance based on a version of ADL-I containing only items displaying fit to the Rasch rating scale model to (b) measures of quality of ADL task performance based on a version of ADL-I containing all 47 items. Similarly, it was investigated whether retaining the items displaying DIF for gender or diagnosis would disrupt the measurement system by means of DTF. By comparing (a) quality of ADL task performance measures based on gender- or diagnosis specific item calibrations with (b) quality of ADL task performance measures based on common item calibrations, the variance of measures across a gender- or diagnosis-specific version and a common version could be explored, for male, females and the six diagnostic subgroups.

Finally, the last research question concerned the precision and reliability of the ADL-I measures. First, it was evaluated whether the mean ADL item difficulty measure was appropriately targeted to the mean quality of ADL task performance measure of the participants. Second, the dispersion of the ADL item difficulty and quality of ADL task performance measures was evaluated for a reasonable match by examining the item-person map, a graphic display of the distribution of item and person measures, generated by the WINSTEPS program. Precision was evaluated by overall separation and reliability indices.

## Results

Data on n = 2198 persons were extracted from the database, based on the inclusion criteria. After removing n = 60 (2.7%) persons with maximum scores on the ADL-I, the study sample used in the initial analyses was n = 2138 persons representing six diagnostic groups (Table 2). Later, after removal of items displaying misfit, data on another n = 40 (1.9%) persons with maximum scores on the ADL-I were removed from the final analyses (n = 2098). Thus, a total of n = 100 persons, of which n = 48 were women, were removed from the analyses representing all diagnostic group (neurologic n = 52; geriatric n = 24; psychiatric n = 14; cancer n = 8; and rheumatologic n = 2), but medical conditions.

**Table 2:** Demographic data

Diagnostic groups	Initial sample	Final sample	Psychiatric	Rheumatologic	Cancer	Medical	Neurologic	Geriatric
	(n = 2138)	(n = 2098)	(n = 142)	(n = 194)	(n = 178)	(n = 116)	(n = 65)	(n = 1403)
Female, n (%)	1447 (67.7)	1433 (63.8)	67 (47.2)	183 (94.3)	89 (50.0)	74 (63.8)	33 (50.8)	987 (70.3)
Age (years), *M* (SD)	73.6 (15.0)	73.7 (14.9)	*42.0 (18.0)	59.2 (16.9)	67.4 (9.7)	76.1 (10.3)	68.8 (9.8)	79.8 (7.9)
Range	19–99	19–99	19–85	21–95	38–89	29–92	44–88	55–99

*SD* Standard deviation

*Mean age is based on the age of n = 140 due to missing data

### Psychometric properties of the rating scale

Initial evaluation of the psychometric properties of the 0–3 rating scale revealed > 10 observations in each category with a frequency peak in category 3 (Table 3). Average category measures advanced monotonically up the rating scale, but thresholds from categories 0 to 1, and 1 to 2 were below 1.4 logits, and threshold disordering was found between categories 1 and 2. All category outfit *MnSq* values were < 2.0 logits. In the final analyses, after removal of misfitting ADL items, category thresholds increased and there was no threshold disordering (Table 3).

**Table 3:** Rating scale category statistics

Rating scale	Frequency	47 ADL-I items (n = 2138)				Frequency	31 ADL-I items (n = 2098)			
		Category measure	Category threshold*	Calibration threshold	Outfit *MnSq*		Category measure	Category threshold*	Calibration threshold	Outfit *MnSq*
0 Unable	9110	− 1.75	None	None	1.64	5904	− 2.06	None	None	1.36
1 Help/safety	7421	− 0.54	*1.21*	− **0.10**	1.10	6033	− 0.66	1.40	− 0.55	1.11
2 Time/effort	20,114	0.44	*0.98*	− **0.46**	1.01	16,122	0.53	*1.19*	− 0.46	0.95
3 Competent	55,454	1.89	1.45	0.56	0.99	35,227	2.26	1.73	1.01	1.00

Bold text indicates threshold disordering. Italic text indicates low category thresholds.

*Category threshold values represent the distance between category measures

### Unidimensionality

The initial PCA of the standardized residuals revealed that 54.3% of the total variance was explained by the Rasch dimension, but the unexplained variance in the first contrast had an eigenvalue of 3.8, suggesting a second dimension with 3–4 items. Nevertheless, disattenuated correlations were above 0.7 between all clusters, supporting unidimensionality.

Still, ten items displayed both high infit and outfit (underfit) misfit: *Calling for attention, Reading, Using the phone, Bowel and urine elimination volitional, Pedicuring, Writing by hand/using word processor, Driving car, Making plans for shopping, Shaving/make-up, Riding bicycle/moped*. Two items only displayed high infit misfit: *Manicuring, Light washing by hand,* and one item displayed only high outfit misfit: *Taking part in a conversation* (Table 4). During the process of removing items with high infit misfit, another three items revealed high infit misfit: *Going by car, Going by bus/tram/tube* and *Going by train/boat/aeroplane,* and were therefore removed. Four items displayed overfit misfit: *Washing body/bathing/showering, Dressing lower trunk, Dressing upper trunk* and *Undressing.* These were retained as they did not pose a threat to the measurement system.

**Table 4:** Items measurement report

ADL-I item	Count	Measure	*SE*	Infit		Outfit	
				*MnSq*	*z*	*MnSq*	*z*
Weekly heavy cleaning (*hardest item*)	2020	2.38	0.03	1.09	2.66	1.16	2.95
Weekly/large quantity shopping^b^	1847	1.80	0.03	1.04	1.36	1.04	0.93
*Pedicuring*	2103	1.75	0.03	**1.81**	**9.90**	**1.96**	**9.90**
*Riding bicyckle/moped*	687	1.60	0.04	**1.51**	**8.71**	**1.53**	**6.76**
Heavy washing in washing machine	1971	1.57	0.03	1.12	4.04	1.08	2.12
Going by train/boat/aeroplane^a^	769	1.39	0.04	1.09	1.87	0.98	− 0.38
Light washing in washing machine	1983	1.32	0.03	1.15	5.05	1.10	2.55
*Driving car*	730	1.19	0.04	**1.63**	**9.90**	**1.56**	**7.22**
Go by bus/tram/tube^a^	1300	1.18	0.03	1.03	0.73	1.01	0.22
Daily/small quantity shopping	2027	1.07	0.03	0.88	− 4.15	0.85	− 3.95
Daily light cleaning^b^	2050	1.04	0.03	0.94	− 1.95	1.02	0.40
Cooking a hot meal	1874	0.99	0.03	1.16	4.86	1.31	6.96
Walking/moving in the neighbourhood	2106	0.75	0.03	0.76	− 8.64	0.83	− 4.40
*Light washing by hand*^*b*^	1354	0.65	0.03	**1.33**	**7.95**	1.20	3.57
Walking/moving from one floor to another	2100	0.56	0.03	0.70	− 9.90	0.79	− 5.14
Washing body/bathing/showering	2120	0.46	0.03	0.58	− 9.90	0.66	− 8.50
Pulling on stockings/pantyhose/shoes	2122	0.36	0.03	0.78	− 7.13	0.94	− 1.27
Washing one’s hair	2116	0.34	0.03	0.91	− 2.92	0.91	− 2.00
Walking/moving in and out of the house	2119	0.26	0.03	0.80	− 6.51	0.76	− 5.26
Going by car^a^	1884	0.22	0.03	1.01	0.29	1.02	0.30
Dressing lower trunk	2129	− 0.01	0.03	0.55	− 9.90	0.64	− 7.71
Preparing a cold meal	2060	− 0.07	0.03	0.89	− 2.85	0.79	− 3.98
Heating up liquid or prepared food	2007	− 0.10	0.03	1.05	1.30	0.90	− 1.84
Transferring the body from bed to chair	2132	− 0.15	0.03	0.72	− 7.89	0.86	− 2.64
*Manicuring*	2107	− 0.15	0.03	**1.38**	**8.73**	1.27	4.36
Walking/moving from one room to another	2130	− 0.19	0.03	0.72	− 7.99	0.77	− 4.40
Undressing	2128	− 0.23	0.03	0.60	− 9.90	0.59	− 8.23
Dressing upper trunk	2126	− 0.24	0.03	0.60	− 9.90	0.64	− 6.91
Transfer in bed, changing positions, turning over, sitting up	2122	− 0.33	0.03	0.82	− 4.59	0.94	− 0.89
*Making plans for shopping*	1999	− 0.48	0.04	**1.57**	**9.90**	**1.54**	**6.85**
*Writing by hand/using word processor*	2105	− 0.52	0.04	**1.41**	**7.98**	**1.73**	**8.91**
Getting necessary clothes from closets/drawers	2114	− 0.62	0.04	0.99	− 0.13	0.72	− 4.45
Getting to and from the toilet room in time	2108	− 0.64	0.04	0.78	− 5.12	0.75	− 3.85
Arranging clothes and equipment, washing hands	2107	− 0.76	0.04	0.70	− 6.86	0.55	− 7.15
Getting on/of toilet and cleaning one’s self after elimination	2115	− 0.77	0.04	0.78	− 4.82	0.68	− 4.84
*Shaving/make-up*	1985	− 0.78	0.04	**1.55**	**9.06**	**1.42**	**4.78**
*Reading*	2112	− 0.78	0.04	**1.68**	**9.90**	**2.47**	**9.90**
Getting food and liquid, cutting up food	2129	− 0.80	0.04	1.05	1.06	0.88	− 1.64
Washing hands and face	2126	− 1.10	0.05	0.84	− 3.05	0.63	− 4.95
*Using the phone*	2118	− 1.27	0.05	**1.47**	**6.89**	**2.07**	**8.83**
*Bowel and urine elimination volitional*	2115	− 1.29	0.05	**1.37**	**5.44**	**2.04**	**8.59**
Combing one’s hair^b^	2109	− 1.29	0.05	1.14	2.22	1.10	1.08
Brushing teeth	2121	− 1.43	0.05	1.00	0.04	1.22	2.11
*Taking part in a conversation*	2121	− 1.51	0.06	1.05	0.73	**1.80**	**8.33**
Eating	2134	− 1.66	0.06	0.98	− 0.21	1.34	2.90
*Calling for attention*	2126	− 1.80	0.06	**1.43**	**5.10**	**2.76**	**9.90**
Drinking, getting liquid from glass into mouth (*easiest item*)	2132	− 1.92	0.07	1.04	0.52	1.31	2.41
Mean	1959.6	0.00	0.04	1.05	0.00	1.15	0.60
SD	362.2	1.07	0.01	0.32	6.30	0.50	5.40

Bold text indicates initially misfitting ADL-I items

^a^Items starting to misfit, after removal of initially misfitting items

^b^Items displaying differential item functioning (DIF) based on gender

**Fig. 1:**
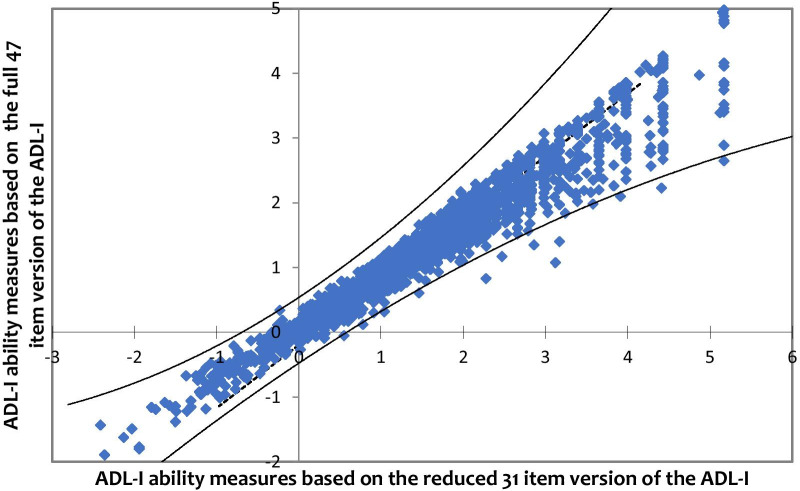
Scatterplot (DTF analysis) of the variance of ability measures across the full (47-item) and reduced (31-item) version of the ADL-I. The model for invariance of measures is represented by a straight line with a slope equal to 1 (i.e. 45°) through the point representing the mean quality of ADL task performance measure from each version. Control lines, representing a 95% confidence interval on either side of the diagonal line, drawn through the mean quality of ADL task performance measures, illustrate quality of ADL task performance measures displaying significant variance across the two versions [23]

After removal of a total of 16 underfitting items, in the PCA the variance explained by the Rasch dimension increased to 58.0%, whereas the eigenvalue representing variance explained by first contrast slightly decreased to 3.5 on the 31-item measurement model. The disattenuated correlations between person measures generated based on three sets of item clusters remained above 0.7. The DTF analysis of the variance of ability estimates across the full (47-item) version of the ADL-I and the 31-item version is illustrated in Fig. 1. The analysis revealed n = 49 (2.3%) participants with significantly different quality of ADL task performance measures between the full version and the 31-item version. Thus, the 16 misfitting items were not a threat to the measurement system.

### Person-response validity

When evaluating person-response validity based on the 47 items in the ADL-I, n = 186 (8.7%) persons did not have ADL-I measures with acceptable goodness-of-fit to the Rasch model. After removal of misfitting items, the proportion of persons with misfitting ADL-I measures increased slightly to 9.1%: psychiatric n = 22 (15.5%); rheumatologic n = 7 (3.6%); cancer n = 25 (14.0%); medical n = 4 (3.5%); neurologic n = 8 (12.3%) and geriatric n = 125 (8.9%).

### Differential item functioning

**Fig. 2:**
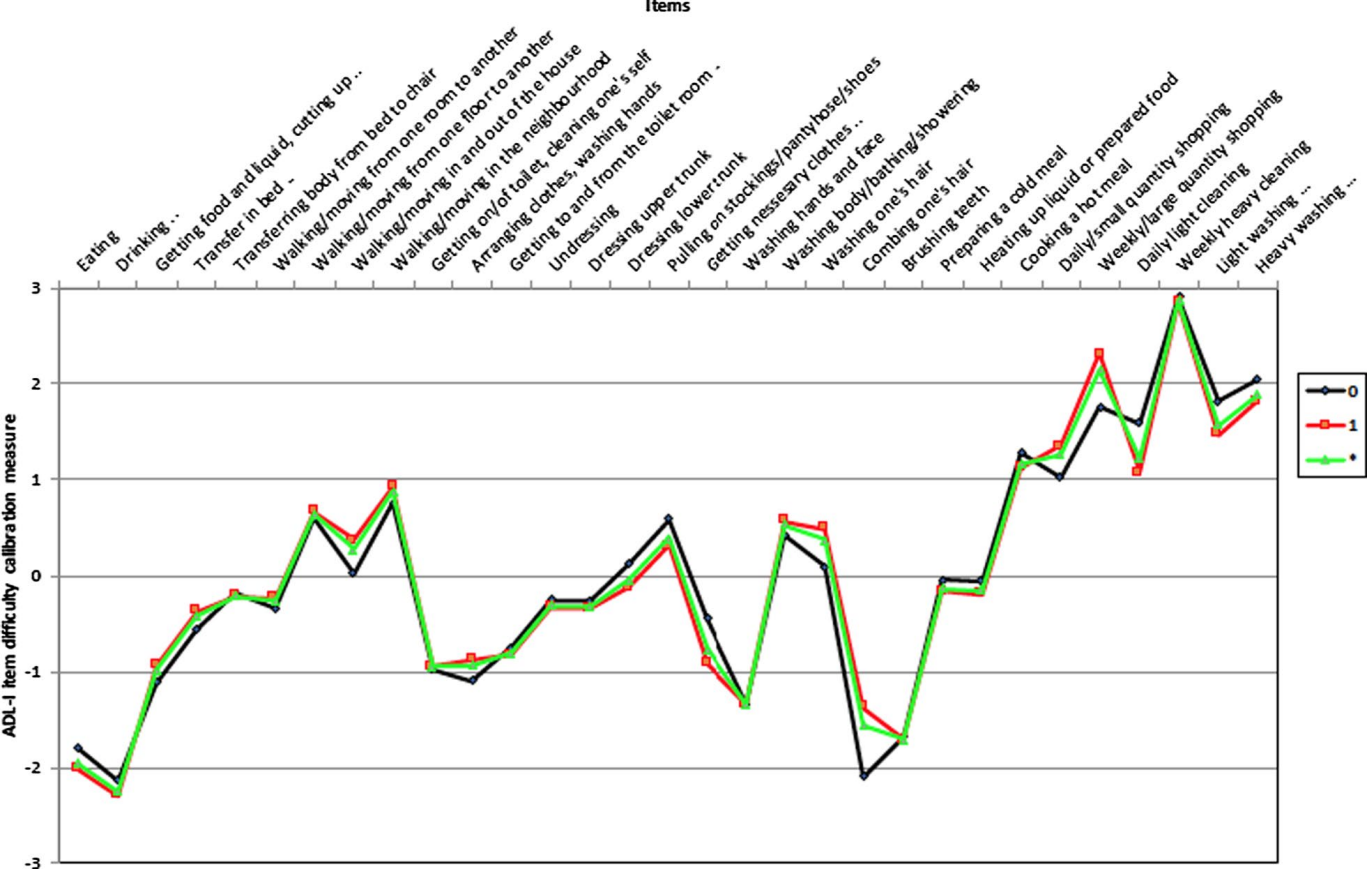
Plot of ADL-I item difficulty calibration measures based on gender. Blue line; item difficulty measures based on male participants. Red line; item difficulty measures based on female participants Green line; average item difficulty measures for both gender groups

Based on the 31-item version of the ADL-I, analysis of DIF based on gender revealed that the items *Combing one’s hair* (p = 0.002) and *Weekly shopping* (p < 0.001) were relatively easier for males and *Daily light cleaning* (p < 0.001) was relatively easier for females (Fig. 2).

**Fig. 3:**
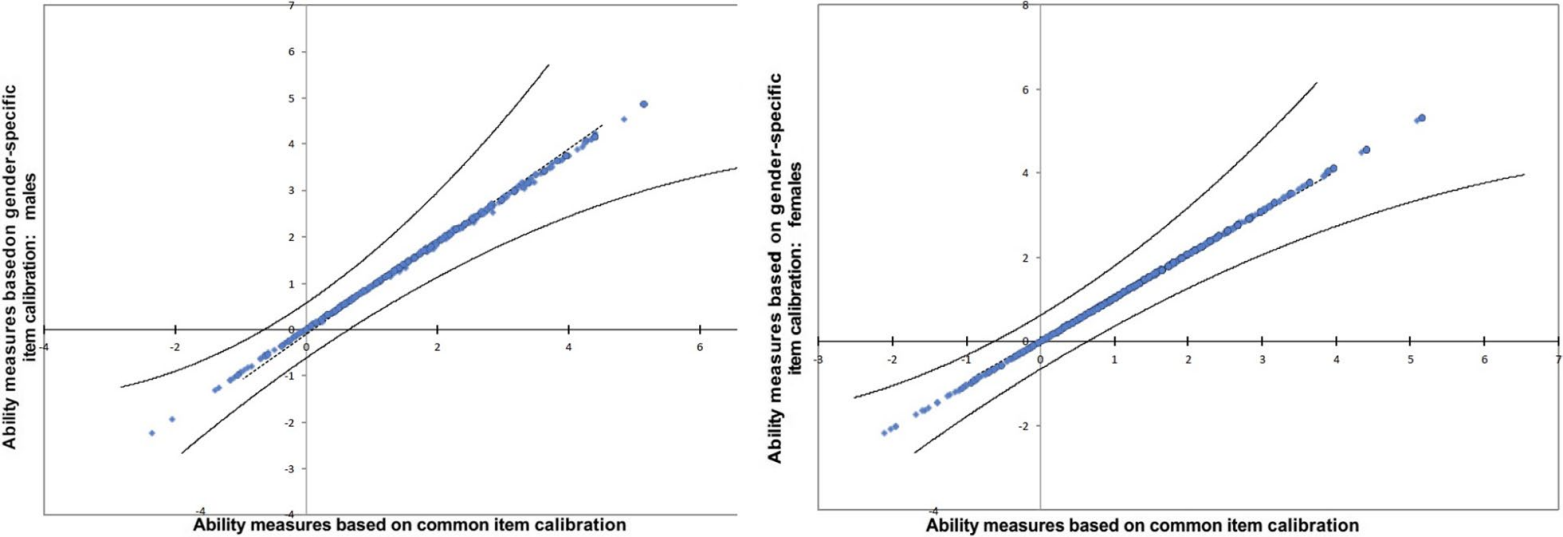
Scatter plot of ADL-I ability measures based on common item calibration and ADL-I ability measures based on gender-specific item calibrations

**Fig. 4:**
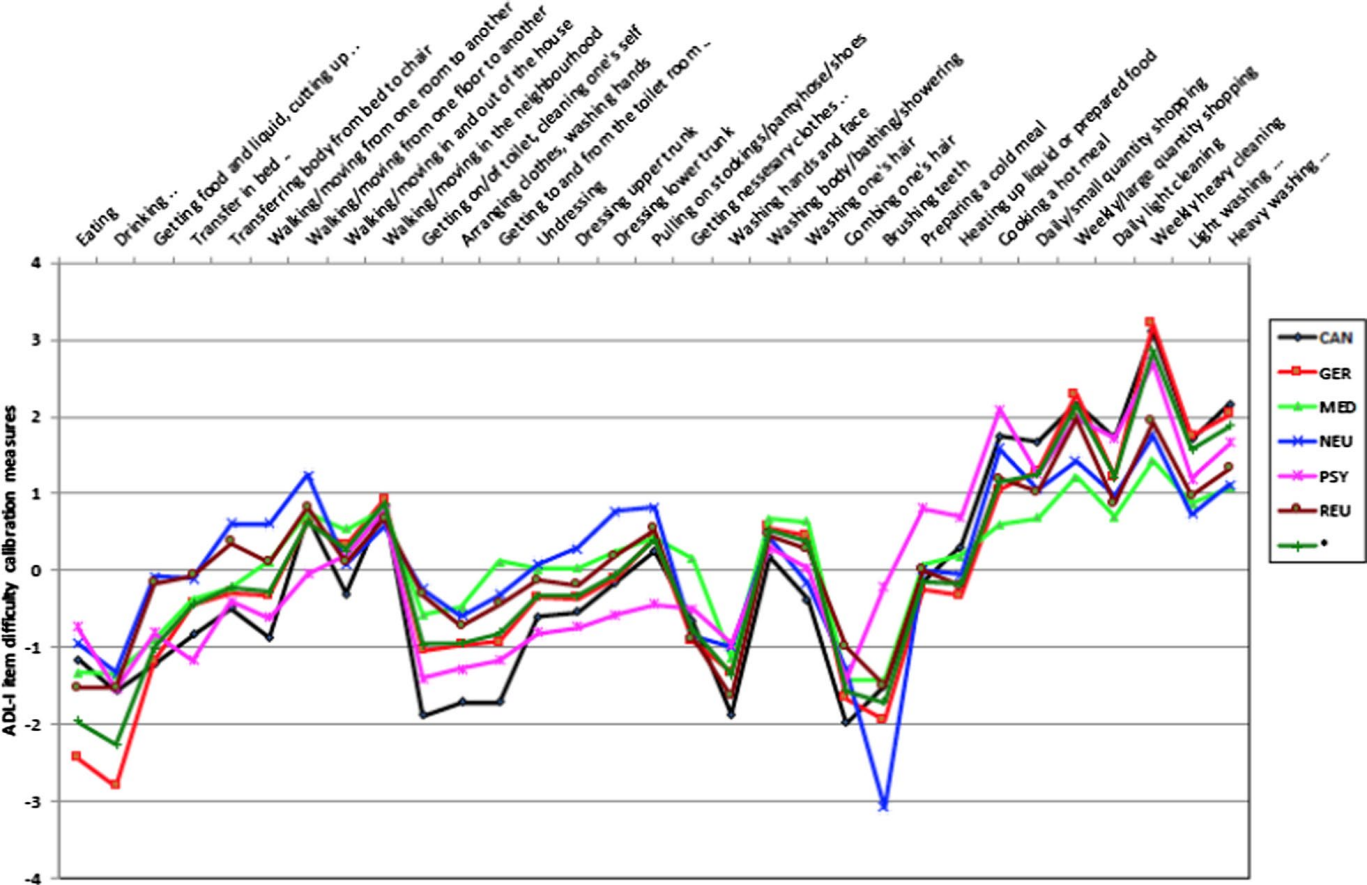
Plot of ADL-I item difficulty calibration measures based on diagnoses

Still, DTF analyses based on scatter plots (Figs. 3 and 4) illustrating the variance of ADL ability measures across the common and gender-specific item calibrations for males and females, respectively, showed that all person ADL ability measures fell within the 95% confidence interval control lines, indicating no significant difference in person ADL ability measures between the common version and the gender-specific versions. Thus, indicating that the items initially displaying uniform DIF were not a threat to the measurement system.

**Fig. 5:**
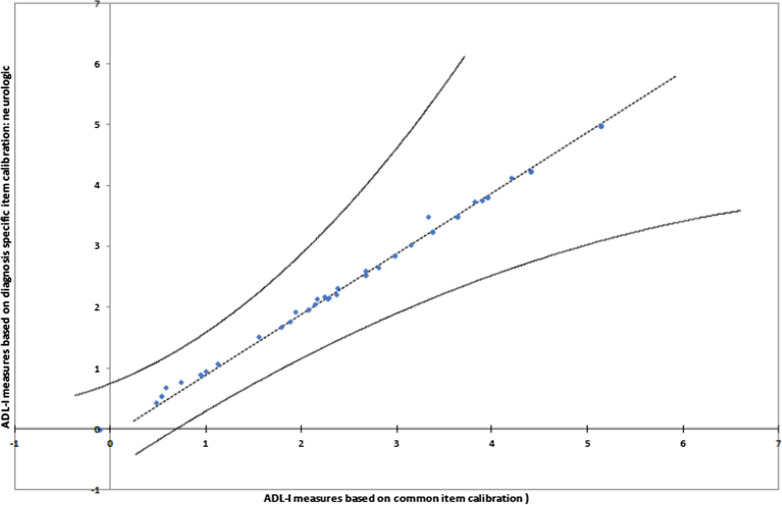
Scatter plot of ADL-I ability measures based on the common item calibration and ADL-I ability measures based on a diagnosis-specific item calibration (neurologic)

DIF analyses based on diagnoses also revealed uniform DIF in relation to some of the items (Fig. 4). Still, patterns similar to DIF by gender were found in DTF analyses using scatter plots of the variance of ADL ability measures across the common and the diagnosis-specific item difficulty calibrations for each of the six diagnostic subgroups. This is illustrated by an example using data on people with a neurologic condition in Fig. 5. Thus, all person ADL ability measures fell within the 95% confidence interval control lines, indicating no significant differences in person ADL ability measures between the common version and the diagnosis-specific versions of the instrument. Hence, the DIF based on diagnoses did not influence the measurement system.

### Reliability and precision

**Fig. 6:**
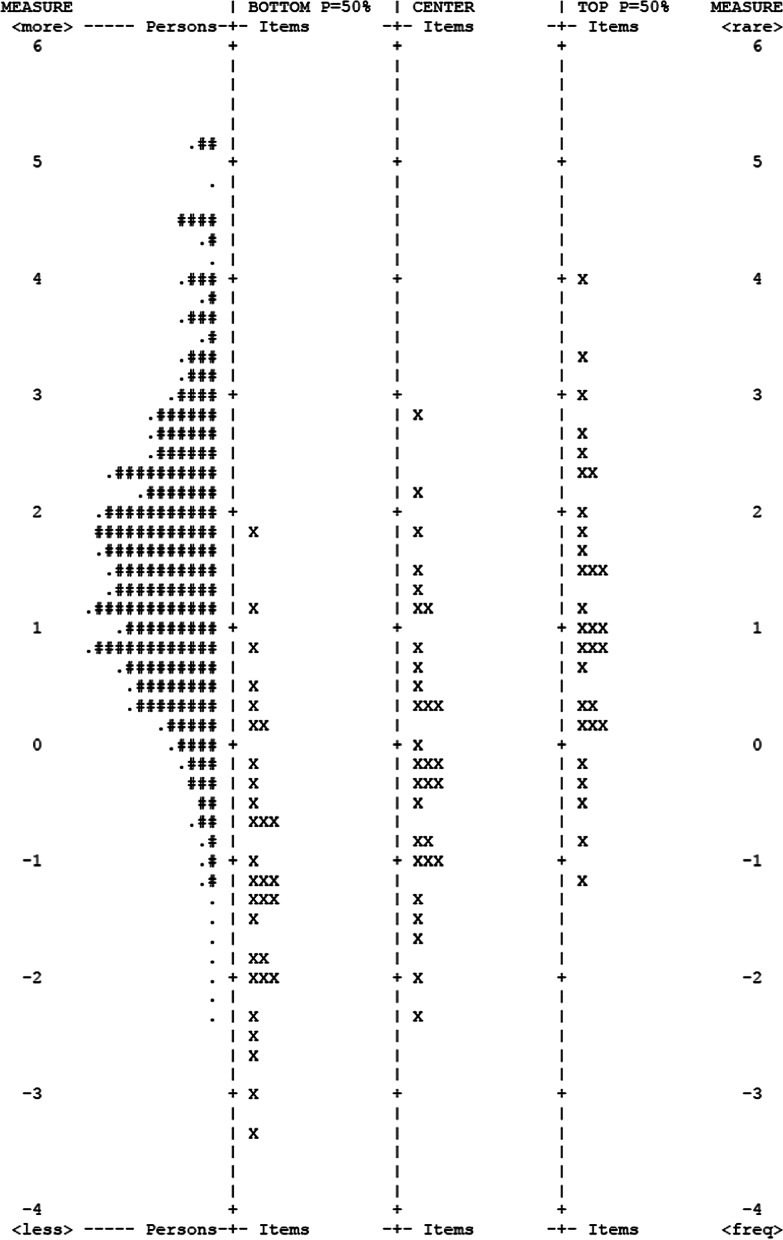
Item-person distribution map—31 item version of the ADL-I. The most difficult items and the most able participants at the top. Each ‘#’ is 10 persons, each ‘.’ is 1 to 9. *Note*: Each item is shown in the three columns representing different rating scale measures. Items: Center = the mean item difficulty calibrations; Items: Bottom = measure level corresponding to a probability of 0.5 of being rated in (or exceeding) the lowest category of the rating scale; Item: Top = measure level corresponding to a probability of 0.5 of being rated in (or falling below) the highest category of the rating scale

The targeting of the 47-item version of the ADL-I to the participants’ ADL ability (mean item difficulty measure: zero, SD 1.06; mean person ADL ability estimate: 1.51, SD 1.31) indicated that the participants had a higher mean level of ADL ability than the mean item difficulty estimate (expected to be zero logits). After removal of misfitting items and participants with maximum scores, the mean item difficulty measure SD increased to 1.22, and the mean ADL ability measure increased to 1.59 (SD decreased to 1.28), indicating slightly diminished targeting of the 31-item version of the ADL-I. The item/person distribution map (see Fig. 6), based on the 31-item version of the ADL-I, illustrates that the items and participants were well distributed along the scale, with item and person measures well targeted to each other, indicating a small ceiling effect and no floor effect.

The initial person separation for the 47-item version of the ADL-I was 2.75 (reliability = 0.88) indicating that the items separated the persons into three significantly different levels of ADL ability [42]. After removal of misfitting items the person separation index improved (2.99; reliability = 0.90).

## Discussions

As the number of persons diagnosed with multi-morbidity is increasing, there is a need for generic instruments to be able to assess, measure and compare ADL ability across gender and diagnoses. Since the ADL-I was developed as a generic instrument to be used across gender and diagnostic groups in rehabilitation research and clinical practice, the aim of this study was to examine the psychometric properties of the ADL-I applied among males and females living with various chronic conditions. While the ADL items displaying misfit overall did not represent a threat to the measurement system, removal of the misfitting items improved rating scale functioning and increased the sensitivity of the ADL-I ability measures. When the misfitting ADL items were removed, the results overall suggested that the ADL-I is producing valid and reliable measures across gender and diagnostic groups among persons within a broad range of ADL ability, providing evidence to support generic use of the ADL-I.

There might be several explanations to the misfitting items revealed in the present study. The majority of items misfitting belonged to two domains: communication and transportation. Items related to communication have been found to misfit in other ADL instruments [16, 44, 45]. In a study involving the ADL-focused Occupation-based Neurobehavioural Evaluation (A-ONE), two communication items: *Comprehension* and *Expression,* were found to misfit [44], and consequently removed from the measurement system in a recent study on the Japanese version of the A-ONE [46]. Similarly, in a Rasch analysis of the Functional Independence Measure (FIM™), similar communication items: *Comprehension* and *Expression*, were reported to misfit [45]. Also, in a Rasch analysis of the ADL-Observation (ADL-O), an instrument similar to the ADL-I, but based on observation, the same five communication items were displaying misfit [16]. Like in the present study, DTF analysis of the ADL-O suggested that the misfitting communication items did not disturb the measurement system, supporting retaining the items in the instrument. Still, the fact that communication items seem to misfit across a range of ADL instruments, suggest that communication represents a dimension not belonging to the concept of ADL, supporting permanent removal of the five ADL-I communication items from the ADL-I.

Items related to transportation have also been found to misfit in other instruments [16, 45]. In the Rasch analysis of the ADL-O, the item of *Driving* did display misfit [16] and in the Instrumental Activity Measure (IAM) the item *Public Transport* was found to misfit [45]. Again, as the DTF analysis suggested that the misfitting transportation items did not influence the measurement system, it can be discussed whether the five transportation items in the ADL-I should be removed or retained.

It is well established that items concerning bowl and bladder functioning often display misfit in traditional ADL scales like the Barthel Index [47, 48] and FIM™ [45, 49, 50]. One explanation could be that bowl and bladder functioning conceptually is addressed as body functioning (continence) rather than the ability to handle actions related to bowel and urine elimination. Hence, the question posed may be focused on whether the person is continent or not, rather than the ability to e.g., schedule frequent toilet visits or handle aids such as incontinence pads or urinals, if incontinent. Since the ability to handle actions related to bowel and urine elimination, being incontinent or not, is important for independent living, the item of *bowel and urine elimination* in the ADL-I should be contained but reformulated to more clearly reflect the intended content.

The remaining five items displaying misfit involve *Shaving/make-up, Manicuring, Pedicuring, Making plans for shopping,* and *Light washing by hand,* all of which seem relevant in an ADL instrument. The items in the ADL-I was originally adopted from the ADL Taxonomy—a classification of ADL tasks in twelve ADL domains, carefully developed in several steps [19, 20]. Still, based on the present results, some of these items may need clarification and reformulation. For example, the item of *Shaving/make-up* covers two separate ADL tasks in one and, along with the items *Making plans for shopping,* and *Light washing by hand,* it represents items not all people do. In terms of the two items concerning nail care, one reason for misfit could be that answers were based on what the person did (regularly visiting e.g., a podiatrist) rather than what he or she was able to perform himself/herself. Since ADL-I is designed to assess the ability to perform ADL tasks in a safe, efficient, effortless and independent manner, it is necessary that the interviewers ask what the person is able to, not what he or she actually does.

The analysis for DIF by gender identified two items (*Combing one’s hair *and *Weekly shopping*) relatively easier for males and one item (*Daily light cleaning*) relatively easier for females. Similar examples of DIF have previously been observed in a study by Fleishman et al. where elderly females were more likely to receive help with shopping than elderly males, whereas elderly males potentially needed more help doing light housework [51]. It has been suggested that these examples of DIF by gender in part can been attributed to historical gender roles [52, 53], explaining how males and females living together typically take charge of different IADL tasks based on roles, habits, routines and preferences. Still, the difficulty of a task may not just be explained by level of routine, but also variations within the task across gender. One example may be that most males prefer a low-maintenance hairstyle, whereas many females wear their hair in a way requirering styling.

Likewise, DIF by diagnoses were identified across the ADL-I items, indicating that item difficulty estimates were sensitive to the characteristics of the diagnostic groups. Still, the DIF based on diagnoses did not influence the measurement system as no significant differences in person ADL ability measures were identified between the common version and the diagnosis-specific versions of the ADL-I. Similar findings have been reported in a study involving the FIM™ employed in two diagnostic groups; stroke and orthopedic impairments [54]. While several items were displaying DIF by diagnoses e.g., eating and bowel continence, minimal influence on FIM measures was identified.

While the ADL-I items displaying misfit or DIF by gender or diagnoses did not influence the overall measurement system, removal of misfitting ADL items improved the rating scale in terms of increased category thresholds and no threshold disordering. Moreover, after removal of almost 1/3 of the initial items, the instrument became more sensitive to detect differences, as indicated by the increased person separation index. One reason could be that most items removed represented other dimensions than ADL e.g., body functions (bowl/bladder), communication and transportation. Still, a few misfitting items do represent the ADL dimension. Thus, other reasons for misfit e.g., unclear definitions of the items should be explored and if possible, resolved. Also, since up to 5% of items on a scale are expected to misfit by chance [55], 1–2 items demonstrating misfit may be kept in the scale.

Since the 47 ADL items of the ADL-I, based on the ADL Taxonomy, previously have been reported as relevant for clinical use, all ADL items can be retained in the ADL-I for the purposes of assessing and describing single client’s quality of ADL task performance, and identifying targets in rehabilitation processes. For measuring self-reported quality of ADL task performance in clinical and research only items displaying fit to the Rasch measurement model should be used. For this purpose, conversion tables can be made available based on the 31-item version. Future studies are needed to evaluate the clinical utility of the ADL-I, including the ease to use in clinical research and practice [56].

### Strength and limitations

The inclusion of a large and diverse study sample representing variation across age, gender, diagnoses and self-reported quality of ADL task performance was a great strength of the Rasch analysis. Moreover, the data was extracted from a research database containing ADL-I data from a range of research studies. Hence, all data were collected for research purposes by trained occupational therapists, supporting data quality. As the database is constructed based on anonymized datasets only including age, gender, diagnosis and raw ADL-I ratings, the study was limited in terms of describing the study sample in more detail. Moreover, the sample sizes for persons with medical, rheumatological, oncological, neurological, and psychiatric chronic conditions may be considered small and potentially result in unstable measures. Still, according to Linacre, a sample size of 64 to 144 persons will provide stable item calibrations and person ability measures within 0.5 logits (CI 95%) [57].

## Conclusions

The 31-item version of the ADL-I is producing valid and reliable measures across gender and diagnostic groups among persons within a broad range of ADL ability.

## Data Availability

The datasets used and/or analysed during the current study are available from the corresponding author on reasonable request.

## Abbreviations

ADL: Activities of daily living
ADL-I: ADL interview
ADL-O: ADL observation
AMPS: Assessment of motor and process skills
A-One: ADL-focused occupation-based neurobehavioural evaluation
COPD: Chronic obstructive pulmonary disease
DIF: Differential item functioning
DTF: Differential test functioning
FIM™: Functional independence measure
IADL: Instrumental activities of daily living
MnSq: Mean square
PADL: Personal activities of daily living
PCA: Principal component analysis
PCM: Partial credit model
RSM: Rasch Rating Scale model
